# Pan-Indian Clinical Registry of Invasive Fungal Infections Among Patients in the Intensive Care Unit: Protocol for a Multicentric Prospective Study

**DOI:** 10.2196/54672

**Published:** 2024-02-16

**Authors:** Anup Kumar Ojha, Venencia Albert, Saurabh Sharma, Vinaykumar Hallur, Gagandeep Singh, Umabala Pamidimukkala, Kh Jitenkumar Singh, Harleen Kaur, Tadepalli Karuna, Jayanthi Savio, Reema Nath, Immaculata Xess, Prashant Gupta, Anjali Shetty, Madhuchhanda Das

**Affiliations:** 1 Indian Council of Medical Research New Delhi India; 2 Indian Council of Medical Research-National Institute of Medical Statistics New Delhi India; 3 All India Institute of Medical Sciences, Bhubaneswar (AIIMS, Bhubaneswar) Bhubaneswar, Odisha India; 4 All India Institute of Medical Sciences, New Delhi (AIIMS-New Delhi) New Delhi India; 5 Nizam's Institute of Medical Sciences Hyderabad, Telangana India; 6 All India Institute of Medical Sciences, Bhopal (AIIMS-Bhopal) Bhopal, Madhya Pradesh India; 7 St John's Medical College and Hospital Bangalore, Karnataka India; 8 Assam Medical College and Hospital Dibrugarh, Assam India; 9 King George's Medical University Lucknow, Uttar Pradesh India; 10 Parmanand Deepchand Hinduja Hospital (PD Hinduja Hospital) Mumbai, Maharashtra India

**Keywords:** mycology, invasive fungal infections, diagnosis, clinical registry, public health, fungal infections, fungal infection, ICU patients, ICU patient, in-patient, in-patients, long-term stay, mortality, mycoses, India, knowledge gaps, epidemiological, epidemiological factor, antifungal resistance, antifungal, descriptive method

## Abstract

**Background:**

Fungal infections are now a great public health threat, especially in those with underlying risk factors such as neutropenia, diabetes, high-dose steroid treatment, cancer chemotherapy, prolonged intensive care unit stay, and so on, which can lead to mycoses with higher mortality rates. The rates of these infections have been steadily increasing over the past 2 decades due to the increasing population of patients who are immunocompromised. However, the data regarding the exact burden of such infection are still not available from India. Therefore, this registry was initiated to collate systematic data on invasive fungal infections (IFIs) across the country.

**Objective:**

The primary aim of this study is to create a multicenter digital clinical registry and monitor trends of IFIs and emerging fungal diseases, as well as early signals of any potential fungal outbreak in any region. The registry will also capture information on the antifungal resistance patterns and the contribution of fungal infections on overall morbidity and inpatient mortality across various conditions.

**Methods:**

This multicenter, prospective, noninterventional observational study will be conducted by the Indian Council of Medical Research through a web-based data collection method from 8 Advanced Mycology Diagnostic and Research Centers across the country. Data on age, gender, clinical signs and symptoms, date of admission, date of discharge or death, diagnostic tests performed, identified pathogen details, antifungal susceptibility testing, outcome, and so on will be obtained from hospital records. Descriptive and multivariate statistical methods will be applied to investigate clinical manifestations, risk variables, and treatment outcomes.

**Results:**

These Advanced Mycology Diagnostic and Research Centers are expected to find the hidden cases of fungal infections in the intensive care unit setting. The study will facilitate the enhancement of the precision of fungal infection diagnosis and prompt treatment modalities in response to antifungal drug sensitivity tests. This registry will improve our understanding of IFIs, support evidence-based clinical decision-making ability, and encourage public health policies and actions.

**Conclusions:**

Fungal diseases are a neglected public health problem. Fewer diagnostic facilities, scanty published data, and increased vulnerable patient groups make the situation worse. This is the first systematic clinical registry of IFIs in India. Data generated from this registry will increase our understanding related to the diagnosis, treatment, and prevention of fungal diseases in India by addressing pertinent gaps in mycology. This initiative will ensure a visible impact on public health in the country.

**International Registered Report Identifier (IRRID):**

DERR1-10.2196/54672

## Introduction

### Background

Public health is seriously threatened by fungal infections, especially in people with immunosuppression and underlying illnesses such as uncontrolled diabetes, influenza, and COVID-19, where they can result in fatal mycoses and elevated death rates [1,2]. Fungal infections manifest in various clinical forms, each with a different severity range, including superficial, cutaneous, subcutaneous, mucosal, and systemic infections. Some fungi that are a part of the microbiota, such as *Candida* spp, can transform into opportunistic pathogens affecting people who are immunocompromised, including those with HIV, patients with cancer receiving chemotherapy, and people on immunosuppressive medications [2-6]. In 2022, the World Health Organization (WHO) aimed to draw attention to the significance of combating fungal infections. There were 3 categories on this list: critical, high, and medium priority. The WHO described it as “the first global effort to systematically prioritize fungal pathogens, considering their unmet research and development needs and perceived public health importance” [7]. Nevertheless, despite the enormous prevalence of fungal propagules in the environment, which makes exposure inevitable, fungal infections are very uncommon in healthy people and animals with strong immune systems compared to bacterial and viral illnesses [8,9]. However, over the past few decades, there has been an increasing trend of persistent and recurrent fungal infections affecting both humans and animals worldwide, which are driven by both true and opportunistic pathogens [8,10-14]. A small but gradually growing number of ubiquitous fungus species that usually pose little harm to humans have emerged as carriers of deadly illnesses, especially in those with compromised immune systems [12]. The natural warmth of a mammalian body has historically constrained fungi’s ability to cause disease [15]. However, many once harmless fungal species have evolved into infectious agents due to climatic changes brought out by human activity [16,17]. Additionally, the recognized geographic ranges of known fungal infections have grown due to the continually rising global temperatures and increased moisture in some areas [18].

To overcome these lacunae and strengthen the diagnosis and research on fungal diseases in India with a focus on high throughput nonculture antigen, antibody, and molecular testing, the Indian Council of Medical Research (ICMR) has established 8 state-of-the-art Advanced Mycology Diagnostic and Research Centers (AMDRCs) in the country under ICMR-MycoNet Task Force Program. This national network of laboratories aims to improve human resources training and enhance fungal diagnostics, clinical care, research, and public health policies. The primary focus of all these centers is to create awareness of different fungal infections among the general population, develop research, cater diagnostic facilities to its catchment areas, and assess the impact of mycology laboratories on patient care and fungal mapping in the country. As an additional component of this Task Force project, it is proposed to develop a Web-based Mycology Inpatient Clinical Registry to perform prospective surveillance of all fungal infections among patients hospitalized at the ICMR AMDRCs in India. It aims to improve the knowledge gaps related to different fungal infections and understand current approaches to the diagnosis and treatment of invasive fungal infections (IFIs).

### Aim and Objectives

The primary aim of the proposed study is to establish a national clinical registry in mycology to overcome the lack of knowledge on epidemiology, clinical course, and pathological characteristics of IFIs for evidence-based decision-making in clinical practice, public health programs and policy in the Indian context. The objectives of this study are to (1) develop advanced diagnostic facilities (culture, serology, molecular, therapeutic drug monitoring [TDM], and antifungal drug sensitivity test [AFST]) for fungal infections and a comprehensive digital clinical registry of IFIs; (2) describe the overall trends of fungal diseases, emerging fungal infections, and antifungal resistance; (3) map the distribution of fungi in the country through regional surveillance; (4) investigate the clinical features, diagnostics, treatment and patient outcomes among intensive care unit (ICU) patients with IFIs; (5) study the contribution of IFIs in the morbidity and mortality caused by different diseases in ICUs; and (6) investigate the clinical features, diagnostics, treatment, and outcomes among patients with chronic subcutaneous infections, such as mycetoma and chromoblastomycosis (neglected tropical diseases) [19].

## Methods

### Case Definition of IFIs

The proposed study will include proven and probable cases of IFIs as per the European Organization for Research and Treatment of Cancer and the Mycoses Study Group Education and Research Consortium (EORTC/MSG; proven cases) or Blot criteria and Modified EORTC/MSG (probable cases) [20-22] (Multimedia Appendix 1).

### Study Design

A hospital-based multicenter, prospective, noninterventional, observational clinical registry with prospective data collection through ICMR AMDRCs was established under the ongoing MycoNet Task Force project. The project duration is 5 years, with staggered entry of clinical sites following a standardized training and onboarding process.

**Figure 1:**
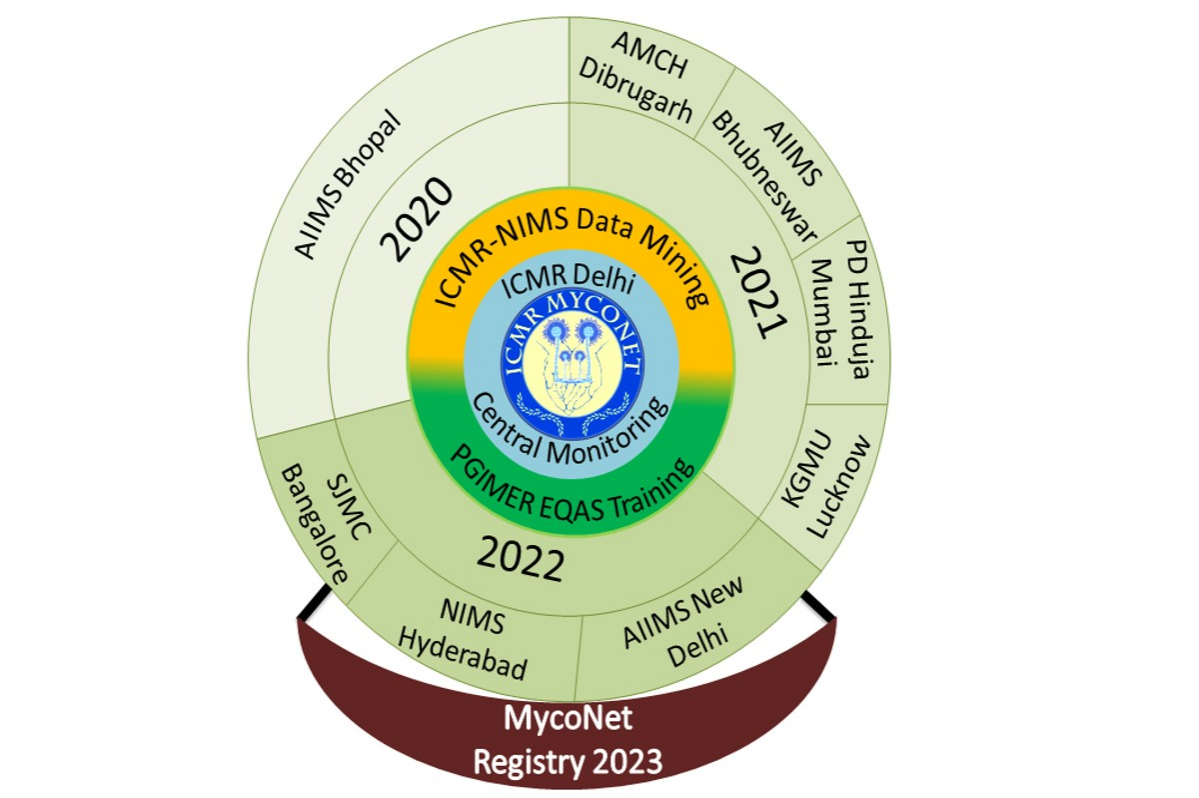
Structural organization of Indian Council of Medical Research (ICMR) Advanced Mycology Diagnostic and Research Centers: 4-tier structure of MycoNet clinical registry. In 2020, 1 site was initiated (All India Institute of Medical Sciences [AIIMS], Bhopal). In 2021, 4 sites were initiated (Assam Medical College and Hospital [AMCH], Dibrugarh; AIIMS, Bhubneswar; P.D. Hinduja, Mumbai; and King George's Medical University [KGMU], Lucknow). In 2022, 3 centers were initiated (St. John's Medical College and Hospital [SJMC], Bangalore; Nizam's Institute of Medical Sciences [NIMS], Hyderabad; and AIIMS, New Delhi). ICMR-NIMS is the data monitoring center; Postgraduate Institute of Medical Education and Research (PGIMER), Chandigarh, is the External Quality Assurance Scheme (EQAS) and Laboratory Training Center; and the ICMR headquarters is the overall coordinator.

This multicentric, Pan-India study was initiated with 5 AMDRCs in 2019. With a scope of increasing the number of reference centers in the next sequential phases of the Task Force project by the expert committee, 3 more centers were introduced and initiated in the financial year 2022-2023. Currently, the network has 8 AMDRCs dispersed across various regions of India. These AMDRCs include (1) All India Institute of Medical Sciences (AIIMS), Bhopal; (2) Assam Medical College and Hospital, Dibrugarh; (3) AIIMS, Bhubaneswar; (4) King George’s Medical University, Lucknow; (5) P.D. Hinduja Hospital, Mumbai; (6) AIIMS, New Delhi; (7) Nizam’s Institute of Medical Sciences, Hyderabad; and (8) St. John’s Medical College, Bengaluru. A timeline for the respective centers with a structural makeup is depicted in Figure 1. To develop a comprehensive network, apart from 8 data collection centers, Postgraduate Institute of Medical Education and Research, Chandigarh, for External Quality Assurance Scheme and laboratory training and ICMR-National Institute of Medical Statistics for data management have been included. ICMR is the overall coordinator of the project. The structural organization of ICMR-MycoNet is depicted in Figure 1. The network offers cutting-edge diagnostic tools for fungi, performs laboratory training to develop trained labor, investigates outbreaks, and conducts research on the epidemiology of fungi and antifungal resistance. The inclusion and exclusion criteria are given in Textbox 1.

**Inclusion and exclusion criteria for MycoNet clinical registry.**

**Inclusion criteria**

- Intensive care unit or hospitalized patients with proven or probable invasive fungal infections confirmed by culture, histopathology, microscopy, or DNA evidence of any age and gender as per European Organization for Research and Treatment of Cancer and the Mycoses Study Group Education and Research Consortium guidelines.
- For individual cases where invasiveness remains unclear, data will be documented, but analysis will be limited to patients with proven and probable invasive fungal infections.
- All patients with a diagnosis of chromoblastomycosis or mycetoma.

**Exclusion criteria**

- Outpatient department or referral patients, patients who may be possible cases of invasive fungal infections, and patients without evidence of invasive disease or those with colonization only will not be included in the registry.
- Additionally, patients with superficial infections, infections limited to the skin, and allergic fungal diseases like allergic bronchopulmonary aspergillosis will be excluded.

Each team consists of a principal investigator from the mycology department and coprincipal investigators from different clinical divisions; including critical care; dermatology; ear, nose, and throat; hematology; infectious disease; internal medicine; and oncology to obtain patient samples. The AMDRCs also work closely with all ICUs, including hemato-oncology, trauma, respiratory, cardiac, and newborn care.

### Data Collection and Management

A case report form (CRF) and standard operating procedures or data entry guidelines were developed. CRF field testing was done to identify the gaps in generated CRF through real-time data recording. Based on the finalized CRF, the electronic CRF was developed using the programming language by ICMR–National Institute of Medical Statistics. Data entry was facilitated through an interactive macrodesigned within the software that can be accessed from any web browser. A digital database will automatically store all the recorded data. The CRF is designed with a drop-down menu to minimize data entry errors. For additional security, a double data entry method will be followed.

The MycoNet Registry app will be maintained on a secure web server at ICMR New Delhi. The hospitals participating in the study will be able to register digitally to initiate the work.

Each AMDRC would receive log-in credentials with access control for data entry, data monitoring, and data download. Data validation will be done by the principal investigator. A dashboard for a snapshot of the data collected from all the AMDRCs (day wise, weekly, or time period) will also be developed for real-time monitoring.

**Figure 2:**
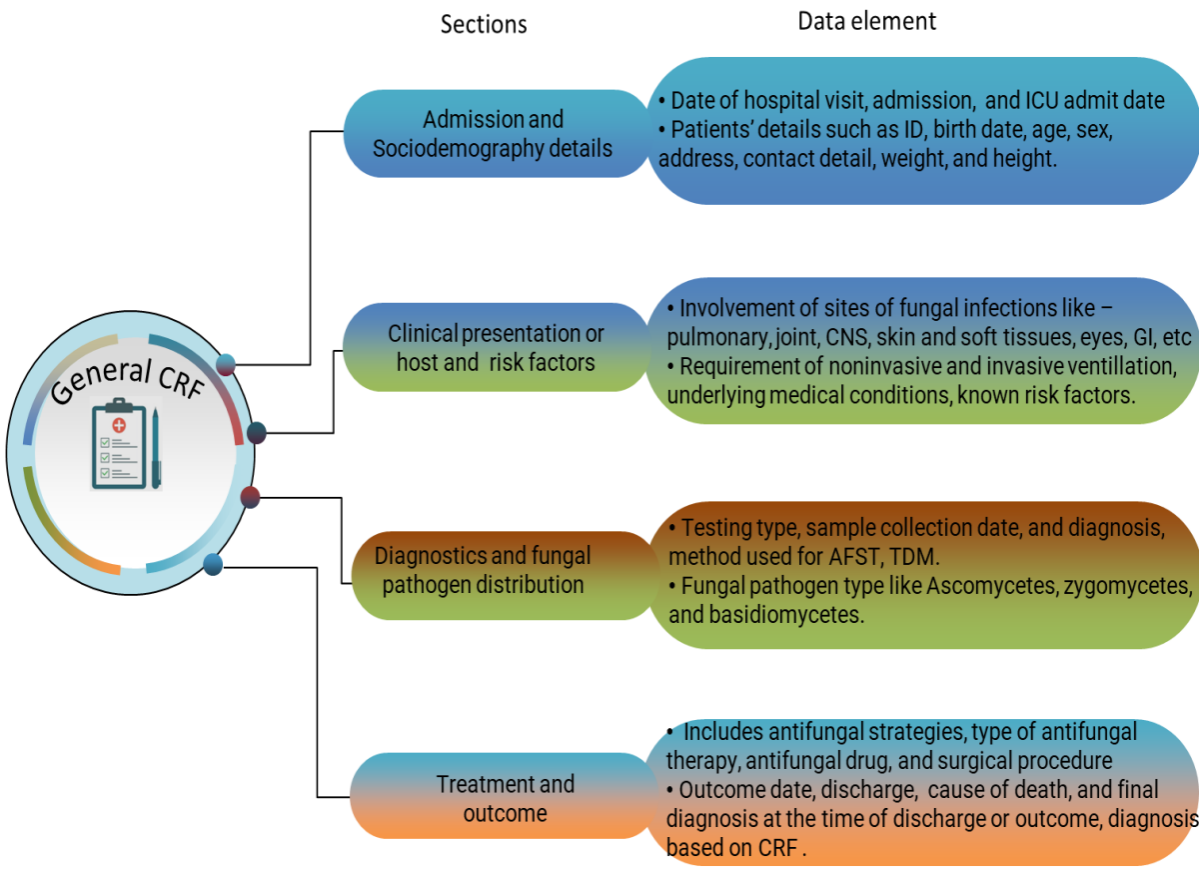
Overview of data variables in the general CRF. AFST: antifungal drug sensitivity test; CNS: central nervous system; CRF: case report form; GI: gastrointestinal; ICU: intensive care unit; TDM: therapeutic drug monitoring.

The data on age, sex, signs, symptoms, date of admission, date of discharge or death, diagnostic tests, identified pathogen, treatment history, AFST report, TDM, any other disease or immunosuppression condition, medical history, outcome, and so on will be obtained from the hospital CRF record. A national fungal strain repository for clinically important and rare fungal strains (yeast and molds) will be developed for future research (Figure 2).

Descriptive analysis of the data will be performed by generating means and proportions. Univariate and multivariate analyses will be undertaken. Further, data analysis will be performed based on the expert committee’s suggestions.

A descriptive analysis of the pattern of clinical presentations by age, sex, and association with risk factors and comorbid conditions across urban and rural regions and states in India will be accomplished. Predictive factors for hospital clinical courses and outcomes will be derived for different treatment settings. The burden of IFIs and associated manifestations will be assessed using standard case definitions and reporting frameworks. Additionally, developing treatment guidelines will be aided by the pattern of comorbidities or risk factors affecting clinical recovery among with patients IFIs.

### Ethical Considerations

There is no interventional aspect to this study. Therefore, there are neither associated risks nor benefits for the patient when participating in the study. The data will be extracted from medical records with no interaction with patients to undertake the secondary data analysis. The study was granted the “waiver of consent,” and ethical approval of the study (CECHR 009/2022) was obtained from the ICMR–Central Ethics Committee on Human Research (reference NCDIR/BEU/ICMR-CECHR/75/2020) as well as the following ethical committees: AIIMS, Bhopal (IHEC-LOP/2021/EF0160); Assam Medical College & Hospital (AMCH), Dibrugarh (2022/AMC/EC/1937); AIIMS, Bhubneswar (T/EMF/Micro/22/38); PD Hinduja Hospital, Mumbai (IRB/1552/AL/22/41); King George’s Medical University (KGMU), Lucknow (1515/Ethics/2022); AIIMS, New Delhi (IEC-695/02.09.2022); Nizam’s Institute of Medical Sciences (NIMS), Hyderabad (EC/NIMS/3038/2022); St John’s Medical College (SJMC), Bangalore (IEC/1/1061/2022) and ICMR–National Institute of Medical Statistics (ICRM-NIMS), New Delhi (11/2022). Memorandums of agreement were signed between the ICMR and all established AMDRCs for shared principles, objectives, and responsibilities. Data security and patient privacy were given the utmost importance throughout the entire process. All information and data extracted from the medical records of the patients for this registry will be considered confidential. The digital documentation of the clinical data will take place in an anonymized fashion. No identifiable data, for example, name or date of birth, will be entered into the database. There will also be no pseudonyms, which would make a retrospective reidentification of the patient possible. Clinical data collected refer to common conditions and treatment modalities in medical care, such that no reidentification of the individual case based on these data will be possible. Any data manipulation by users and administrators will be logged into an audit trail allowing complete data reconstruction. All data and results will be stored for at least 10 years after the publication of the results.

Administration of the electronic CRF will be limited to selected and named administrators at AMDRCs, who will receive comprehensive training in the system before access. Secure passwords are also enforced for administrators, and they must regularly change their passwords.

## Results

This study has been funded by the ICMR. Data collection began in January 2023 in all 8 AMDRCs study sites, after approval from the central and local ethics committee in June 2022. The registry has also been registered with the Clinical Trial Registry–India (registration CTRI/2022/09/045489). To date, 531 cases of IFI, 29 cases of mycetoma, and 1 case of chromoblastomycosis were enrolled in the registry. First-year data analysis has been initiated to be published by the second quarter of 2024. Data from the study are expected to improve the knowledge gaps related to different fungal infections and understand current approaches to diagnosing and treating IFIs. The trends of IFIs, AFST patterns, and the contribution of IFIs to total ICU mortality will be the most valuable outcome of this project. Data on rare fungal diseases such as mycetoma and chromoblastomycosis will be obtained.

## Discussion

### Principal Findings

ICMR has set up a network of laboratories established to address the long need for modern facilities for fungal diagnostics, antifungal resistance mapping, and advanced fungal research in the different geographical regions of the country. Through the ICMR-MycoNet, the generation and systematic collection of robust comprehensive data on IFIs in ICU patients have the potential to fill in important knowledge gaps and guide evidence-based decision-making in clinical practice, public health efforts, and policy reforms in India.

The proposed study has great potential for filling critical knowledge gaps regarding invasive fungal diseases and their effects on public health. A national clinical registry in mycology is being established to lay a solid platform for evidence-based decision-making in clinical practice, public health efforts, and policy creation. The main goals include creating a multicenter digital clinical registry, a thorough examination of epidemiological variables, research into clinical issues, and monitoring of emerging fungal disease trends, which are essential steps toward gaining a thorough understanding of this significant health care issue. Additionally, achieving the secondary goals, which include analyzing the role of fungal infections in morbidity and inpatient mortality across a range of diseases and identifying emerging antifungal resistance patterns, will undoubtedly improve our capacity to control and treat these infections successfully. We can learn a lot more about IFIs from this work, which could greatly impact health care plans and policy.

### Impact of AMDRCs

AMDRC is a new initiative of ICMR headquarters in Delhi to support the establishment of AMDRCs in different geographical regions of the country. Mycology has been one of the neglected areas of research in India. Therefore, advanced diagnostic services and research are required in addition to the cutting-edge research and training of health care professionals to combat IFIs. AMDRCs have considerably increased the diagnostic capacity for fungi illnesses, with a notable improvement in accuracy that has reached up to 95%. The increased diagnosis accuracy ensures that patients get the right care when needed. The AMDRCs have attained a remarkable 95% to 100% detection rate of fungal infections. This lowers the risk of poor management because the majority of cases that were previously undetected are now effectively diagnosed. One of the outstanding advantages of AMDRCs is the free diagnostic services they offer. As a result, patients no longer need to rely on expensive private laboratories for fungal testing, thereby improving access to and affordability of treatment. With AMDRCs’ better diagnostic capabilities, referring patients for identification to higher tier medical centers is no longer necessary. This eliminates the workload on tertiary health care facilities, speeds up the diagnostic process, and decreases treatment delays. AMDRCs have significantly reduced the time it takes to diagnose fungal diseases. It now just takes a few weeks to complete what used to take many months. Clinicians can start prompt and accurate therapy because of the quick reporting, which eventually leads to better patient outcomes. Programs for antifungal stewardship have been made easier by the development of AMDRCs. Clinicians are now better equipped to make decisions about antifungal treatment, and ensuring judicious use of these drugs is a vital step in preventing antifungal resistance. The ability of AMDRCs to provide real-time diagnostic reports has reduced the need for empiric antifungal treatment. By adjusting therapies based on precise fungal identifications, clinicians can lower the likelihood of overmedicating. Drug toxicity is decreased, and therapeutic efficacy is increased due to AMDRCs’ integration of TDM. With this personalized approach, patients are assured of receiving the best antifungal care with minimal side effects. Apart from helping diagnose fungal diseases, adding *Aspergillus* antigen or antibody testing to AMDRC services benefits the overall health care system. These tests demonstrate the adaptability and significance of AMDRCs beyond the control of fungal illness by supporting tuberculosis elimination programs. Thus, this study will be able to address a long-neglected public health issue. However, to get access to diagnostics and antifungal treatment, the study may face regional limitations due to the exclusion of some territories or countries with a hostile environment, poor communication, and fewer populations [23,24].

### Future Plan

#### WHO on Global Antimicrobial Resistance Surveillance System Fungi Program (Global Collaborative Effort to Compile Available Data on IFIs)

The goal of this program is to compile a complete database of information on these diseases, including data concerning the various types of fungi causing the infections, the geographical areas where they tend to be more prevalent, the patterns of antifungal resistance, and other pertinent aspects. By gathering this information, the WHO and its collaborators expect to comprehend better and address the problems caused by IFIs and antifungal resistance.

#### Extended Multicenter Registry Development to Obtain More Validated Data

The purpose of expanding the registries is to include more centers to produce a more robust and extensive database. This is in the context of gathering information on invasive fungal diseases from a wider geographical area and multiple health care facilities for the study. Data gathered from multiple centers will enhance the validity and authenticity of the information.

#### Collaborative Approach

In collaboration with other data sharing registries, researchers can find patterns, trends, and potential treatments for fungal infections by pooling their data more efficiently. Similar to other registries, it encourages teamwork in problem-solving and may result in more efficient diagnosis, treatment, and prevention approaches [25,26]. For surveillance of neglected tropical diseases, a database of mycetoma and chromoblastomycosis will help to identify different nodal areas for future surveillance through active case searches.

#### One Health Approach

Fungi are ubiquitous. Their interaction with agriculture and animals is certain. As part of the “One Health” concept, keeping in view the data of drug-resistant fungi reported through the registry, we can further collaborate with the Indian Council of Agricultural Research and Indian Veterinary Research Institute (both under the Ministry of Agriculture and Farmer Welfare) to study the effect of use of different fungicides in agriculture and animals and study the transmission dynamics within these.

### Conclusions

IFIs are hidden ICU killers. Unfortunately, in a tropical country such as India with a high potential population for fungal infections, mycology is still a neglected area. The present recommendations mostly rely on a compilation of case reports, studies from specific medical facilities, and professional judgments. Analyses must be performed on a large cohort of patients to make treatment recommendations based on sound evidence. It is imperative to explore the contribution of opportunistic fungal infections in the morbidity and mortality of immunocompromised patients and determine the frequency of occurrence of this condition in the country by establishing a registry. Therefore, the development of a Mycology Clinical Registry through the established ICMR-MycoNet centers is proposed to improve the lack of knowledge on epidemiology, clinical course-biology, pathology mechanisms of IFIs and their trends, and AFST and to aid in facilitating an evidence-based diagnostic therapeutic integrated approach to IFI. Antifungal stewardship through this unique initiative will help to reduce empirical antifungal therapy. Any emerging threats, such as the spread of resistant strains or outbreak signals, can be picked up quickly through this real-time database for quick action and policy decisions. These state-of-the-art facilities will be the game changers and able to find cryptic fungal cases successfully, which will visibly change the public health profile of the country.

## Appendix

Multimedia Appendix 1 Guidelines for invasive fungal infection (IFI) diagnosis for respective case definitions.

## Abbreviations

AFST: antifungal drug sensitivity test
AIIMS: All India Institute of Medical Sciences
AMDRC: Advanced Mycology Diagnostic and Research Center
CRF: case report form
EORTC/MSG: European Organization for Research and Treatment of Cancer and the Mycoses Study Group Education and Research Consortium
ICMR: Indian Council of Medical Research
ICU: intensive care unit
IFI: invasive fungal infection
TDM: therapeutic drug monitoring
WHO: World Health Organization

## Data Availability

The data sets used or analyzed during the study will be made available from the corresponding author on reasonable request.

## References

[1] Ray A, Aayilliath KA, Banerjee S, Chakrabarti A, Denning DW (2022). Burden of serious fungal infections in India. Open Forum Infect Dis.

[2] Reddy GKK, Padmavathi AR, Nancharaiah YV (2022). Fungal infections: pathogenesis, antifungals and alternate treatment approaches. Curr Res Microb Sci.

[3] Riley MMS (2021). Invasive fungal infections among immunocompromised patients in critical care settings: infection prevention risk mitigation. Crit Care Nurs Clin North Am.

[4] Clark C, Drummond RA (2019). The hidden cost of modern medical interventions: how medical advances have shaped the prevalence of human fungal disease. Pathogens.

[5] Rajasingham R, Smith RM, Park BJ, Jarvis JN, Govender NP, Chiller TM, Denning DW, Loyse A, Boulware DR (2017). Global burden of disease of HIV-associated cryptococcal meningitis: an updated analysis. Lancet Infect Dis.

[6] Badiee P, Hashemizadeh Z (2014). Opportunistic invasive fungal infections: diagnosis and clinical management. Indian J Med Res.

[7] (2018). WHO fungal priority pathogens list to guide research, development and public health action. World Health Organization.

[8] Gnat S, Łagowski D, Nowakiewicz A, Dyląg M (2021). A global view on fungal infections in humans and animals: opportunistic infections and microsporidioses. J Appl Microbiol.

[9] Pathakumari B, Liang G, Liu W (2020). Immune defence to invasive fungal infections: a comprehensive review. Biomed Pharmacother.

[10] Bishnoi A, Vinay K, Dogra S (2018). Emergence of recalcitrant dermatophytosis in India. Lancet Infect Dis.

[11] Gnat S, Łagowski D, Nowakiewicz A, Osińska M, Kopiński L (2020). Population differentiation, antifungal susceptibility, and host range of trichophyton mentagrophytes isolates causing recalcitrant infections in humans and animals. Eur J Clin Microbiol Infect Dis.

[12] Köhler JR, Casadevall A, Perfect J (2014). The spectrum of fungi that infects humans. Cold Spring Harb Perspect Med.

[13] Köhler JR, Hube B, Puccia R, Casadevall A, Perfect JR (2017). Fungi that infect humans. Microbiol Spectr.

[14] Shenoy MM, Jayaraman J (2019). Epidemic of difficult-to-treat tinea in India: current scenario, culprits, and curbing strategies. Arch Med Health Sci.

[15] Casadevall A (2020). Climate change brings the specter of new infectious diseases. J Clin Invest.

[16] de Crecy E, Jaronski S, Lyons B, Lyons TJ, Keyhani NO (2009). Directed evolution of a filamentous fungus for thermotolerance. BMC Biotechnol.

[17] Casadevall A, Kontoyiannis DP, Robert V (2019). On the emergence of candida auris: climate change, azoles, swamps, and birds. mBio.

[18] Friedman DZP, Schwartz IS (2019). Emerging fungal infections: new patients, new patterns, and new pathogens. J Fungi (Basel).

[19] (2017). Control of neglected tropical diseases. World Health Organization.

[20] De Pauw B, Walsh TJ, Donnelly JP, Stevens DA, Edwards JE, Calandra T, Pappas PG, Maertens J, Lortholary O, Kauffman CA, Denning DW, Patterson TF, Maschmeyer G, Bille J, Dismukes WE, Herbrecht R, Hope WW, Kibbler CC, Kullberg BJ, Marr KA, Muñoz P, Odds FC, Perfect JR, Restrepo A, Ruhnke M, Segal BH, Sobel JD, Sorrell TC, Viscoli C, Wingard JR, Zaoutis T, Bennett JE (2008). Revised definitions of invasive fungal disease from the European Organization for Research and Treatment of Cancer/Invasive Fungal Infections Cooperative Group and the National Institute of Allergy and Infectious Diseases Mycoses Study Group (EORTC/MSG) consensus group. Clin Infect Dis.

[21] Blot SI, Taccone FS, Van den Abeele AM, Bulpa P, Meersseman W, Brusselaers N, Dimopoulos G, Paiva JA, Misset B, Rello J, Vandewoude K, Vogelaers D (2012). A clinical algorithm to diagnose invasive pulmonary aspergillosis in critically ill patients. Am J Respir Crit Care Med.

[22] Donnelly JP, Chen SC, Kauffman CA, Steinbach WJ, Baddley JW, Verweij PE, Clancy CJ, Wingard JR, Lockhart SR, Groll AH, Sorrell TC, Bassetti M, Akan H, Alexander BD, Andes D, Azoulay E, Bialek R, Bradsher RW, Bretagne S, Calandra T, Caliendo AM, Castagnola E, Cruciani M, Cuenca-Estrella M, Decker CF, Desai SR, Fisher B, Harrison T, Heussel CP, Jensen HE, Kibbler CC, Kontoyiannis DP, Kullberg BJ, Lagrou K, Lamoth F, Lehrnbecher T, Loeffler J, Lortholary O, Maertens J, Marchetti O, Marr KA, Masur H, Meis JF, Morrisey CO, Nucci M, Ostrosky-Zeichner L, Pagano L, Patterson TF, Perfect JR, Racil Z, Roilides E, Ruhnke M, Prokop CS, Shoham S, Slavin MA, Stevens DA, Thompson GR, Vazquez JA, Viscoli C, Walsh TJ, Warris A, Wheat LJ, White PL, Zaoutis TE, Pappas PG (2020). Revision and update of the consensus definitions of invasive fungal disease from the European Organization for Research and Treatment of Cancer and the Mycoses Study Group Education and Research consortium. Clin Infect Dis.

[23] Salmanton-García J, Au WY, Hoenigl M, Chai LYA, Badali H, Basher A, Brockhoff RA, Chen SCA, Chindamporn A, Chowdhary A, Heath CH, Jabeen K, Lee J, Matar M, Taj-Aldeen SJ, Tan BH, Uno K, Wahyuningsih R, Zhu L, Chakrabarti A, Cornely OA (2023). The current state of laboratory mycology in Asia/Pacific: a survey from the European Confederation of Medical Mycology (ECMM) and International Society for Human and Animal Mycology (ISHAM). Int J Antimicrob Agents.

[24] Tan BH, Chakrabarti A, Patel A, Chua MMM, Sun PL, Liu Z, Rotjanapan P, Li R, Wahyuningsih R, Chayakulkeeree M, Chen YC (2020). Clinicians' challenges in managing patients with invasive fungal diseases in seven Asian countries: an Asia Fungal Working Group (AFWG) survey. Int J Infect Dis.

[25] Salmanton-García J, Busca A, Cornely OA, Corradini P, Hoenigl M, Klimko N, Marchesi F, Pagliuca A, Passamonti F, Koehler P, Pagano L (2021). EPICOVIDEHA: a ready to use platform for epidemiological studies in hematological patients with COVID-19. Hemasphere.

[26] Koehler P, Arendrup MC, Arikan-Akdagli S, Bassetti M, Bretagne S, Klingspor L, Lagrou K, Meis JF, Rautemaa-Richardson R, Schelenz S, Hamprecht A, Koehler FC, Kurzai O, Salmanton-García J, Vehreschild JJ, Alanio A, Alastruey-Izquierdo A, Arsenijevic VA, Gangneux JP, Gow NAR, Hadina S, Hamal P, Johnson E, Klimko N, Lass-Flörl C, Mares M, Özenci V, Papp T, Roilides E, Sabino R, Segal E, Talento AF, Tortorano AM, Verweij PE, Hoenigl M, Cornely OA (2019). ECMM CandiReg-a ready to use platform for outbreaks and epidemiological studies. Mycoses.

