# Supplementary material for: Pan-Indian Clinical Registry of Invasive Fungal Infections Among Patients in the Intensive Care Unit: Protocol for a Multicentric Prospective Study
Source: JMIR Res Protoc. 2024 Feb 16;13:e54672. doi: 10.2196/54672 (PMC10907932; doi:10.2196/54672)
Supplement: Multimedia Appendix 1 [file resprot_v13i1e54672_app1.docx]

| **S. No.** | **Category** | **Causative agent** | **Criteria** | **Details** | **References** |
| --- | --- | --- | --- | --- | --- |
| 1 | Proven | For all IFIs | EORTC/MSG criteria, 2008 | Annexure II | [20] |
| 2 | Probable  (immunocompromised patients only) | Invasive Pulmonary Aspergillosis (IPA) | Blot criteria 2012 | Annexure III | [21] |
|  |  | Other clinical forms of Aspergillosis or other Moulds or Yeasts or Yeast like fungi | Modified EORTC/MSG based on Blot’s clinical algorithm (Compatible clinical/imaging/mycology findings in different host populations) | Annexure IV | [22] |

**EORTC/MSG criteria, 2019**

| **Causative agent** | **Requirement** | **Details** |
| --- | --- | --- |
| IPA  or  Other molds | - Sterile material - Needle aspiration or sterile Lung biopsy or blood | **1. Histopathologic, cytopathologic, or direct microscopic** examination of a specimen in which hyphae are seen accompanied by evidence of associated tissue damage.  **2. Culture on sterile material,** excluding BAL fluid, a paranasal or mastoid sinus cavity specimen, and urine  **3. Blood culture** for *Fusarium* species only  **4. Serology**: Not applicable  **5. Molecular diagnosis:** PCR +ve with DNA Sequencing along when hyphae seen in formalin fixed paraffin embedded sections (FFPE) |
| Candidemia  or  Cryptococcosis  or  Yeast like fungi | Sterile specimens/ Blood/CSF | **1. Histopathologic, cytopathologic, or direct microscopic examination:** Sterile specimens obtained by needle aspiration or biopsy from a normally sterile site (other than mucous membranes) showing budding yeast cells or with pseudo hyphae/hyphae  **2. Recovery of a yeast by culture of a sample obtained by a sterile procedure** (including a freshly placed [<24 hours ago] drain) from a normally sterile site  **3. Blood culture** that yields yeast (eg, *Cryptococcus* or *Candida* species) or yeast-like fungi (eg, *Trichosporon*species)  **4. Serology:** Cryptococcal antigen in cerebrospinal fluid or blood  **5. Molecular diagnosis:** PCR +ve with DNA Sequencing along when yeasts are seen in FFPE |
| **Pneumocystis** |  | **Detection of the organism microscopically in tissue, BAL fluid, expectorated sputum** using conventional GMS staining or immunofluorescence staining  **Culture/Serology/Molecular:**  Not applicable |
| **Endemic**  **mycoses** |  | **Histopathology or direct microscopy** of specimens obtained from an affected site showing the distinctive form of the fungus  **Culture:** Recovery by culture of the fungus from specimens from an affected site Blood culture that yields the fungus  **Serology/Molecular diagnosis:** Not applicable |

**Blot criteria 2012**

| **Causative agent** | **Requirement** | **Details** |
| --- | --- | --- |
| **Probable IPA** | - **All 4 condition should be meet** i.e**.,**   1. Mycological criteria  2. Radiology  3. Clinical presentation  4. Host factors  (underlying conditions)   - Higher likelihood of Probable IPA if all 4 condition meet, - however, in absence of disease severity /underlying condition suggestive mycology finding and radiologic feature should not be neglected in diagnosis of IPA | **1. Mycological criteria (any of the direct or indirect test positive)**  **Entry criteria**   1. **Direct test (either microscopy or culture positive or both)**  - Respiratory tract samples (preferably lower tract specimens) i.e. BAL/ ET aspirate / Sputum etc. - Cytology/Direct microscopy – Positive - Culture (Qualitative/Semi-quantitative) -Positive  1. **Indirect tests**  - Serum: GM-positive (>0.5 or higher) - BAL fluid: GM-positive (>0.5 or higher) - LFD-test: positive - Aspergillus PCR positive |
|  |  | **2. Radiological features (any one of the following)**   - Dense, well circumscribed lesion(s) with or without a halo sign - Air-crescent sign - Cavity formation - ARDS like images or - nonspecific infiltrates - consolidation - Abnormal imaging on chest X-ray |
|  |  | **3. Clinical presentation (any one of the following)**   - Severe respiratory failure and need for extra-corporal membrane oxygenation (ECMO) - Influenza/Covid-19 or other viral infection - Fever refractory to at least three days of appropriate antibiotic therapy - Recrudescent fever after a period of defervescence of at least 48 h while still on antibiotics and without other apparent cause - Pleuritic chest pain - Pleuritic rub - Dyspnea - Hemoptysis - Worsening respiratory insufficiency in spite of appropriate antibiotic therapy and ventilatory support - Sepsis/multi-organ failure - ARDS - Pneumonia - Higher SOFA (>5)/Apache score |
|  |  | **4. Host factors/underlying conditions (One of the following):**  **Immunocompromised state**   - Neutropenia, - Haematological or oncological malignancy - Glucocorticoid treatment, - Congenital or - Acquired immunodeficiency syndrome   **Other underlying conditions**   - Hepatic cirrhosis - HIV/AIDS - Chronic obstructive pulmonary disease (COPD) - Chronic alcohol abuse - Diabetes mellitus, - Chronic heart failure, - Chronic renal failure - Haemodialysis etc. |

**Modified EORTC/MSG based on Blot’s clinical algorithm**

| **Causative agent** | **Requirement** | **Details** |
| --- | --- | --- |
| Other clinical forms of Aspergillosis  or  other Molds  or  Yeasts  or  Yeast like fungi | - Presence of all of the following, as described by Blot et.al. 2012, 2019 but not limited. - Variable with Causative agent, clinical disease presentations | 1. **Risk factors** as described by Blot et.al. 2012, 2019 but not limited 2. **Compatible clinical features**including acute localized pain (including pain radiating to the eye), Nasal ulcer with black eschar, Focal lesions on imaging etc. 3. **Imaging:** as described by Blot et.al. but not limited including mycotic aneurism, bird’s nest sign, multiple large nodule, pneumothorax, bull’s-eye lesions in liver/spleen/brain etc. 4. **Mycology evidence:**     1. **Either Direct microscopy or culture positive** (preferably both) from **any non-sterile sites**    2. **Serology: β-D-glucan** (Fungitell) ≥80 ng/L (pg/mL) detection in **>2 consecutive serum samples** provided other etiologies have been excluded (for Candida/pneumocystis) |
